# Ultrasound quantitative characterization of tendinopathy with shear wave elastography in an *ex vivo* porcine tendon model

**DOI:** 10.1186/s41747-024-00542-1

**Published:** 2025-03-20

**Authors:** Quinn Steiner, Albert Wang, Laura Slane, Scott Hetzel, Ryan DeWall, Darryl Thelen, Kenneth Lee

**Affiliations:** 1 Department of Radiology, University of Wisconsin-Madison, Madison, WI USA; 2 Department of Biomedical Engineering, University of Wisconsin-Madison, Madison, WI USA; 3 Department of Mechanical Engineering, University of Rochester, Rochester, NY USA; 4 DePuy Synthes, Zuchwil, Switzerland

**Keywords:** Collagenases, Elasticity imaging techniques, Swine, Tendinopathy, Ultrasound

## Abstract

**Background:**

Early detection and treatment of tendinopathy may prevent progression to partial tears or complete rupture. Shear wave elastography (SWE) may help address the need for better tendon pathology characterization. This study aimed to quantify the effect of structural damage in an *ex vivo* animal tendinopathy model using SWE.

**Methods:**

Forty-two porcine flexor tendons were injected with a 0.05-mL bolus of 1.5% collagenase solution to induce focal structural damage without surface tears. Control tendons were injected with saline (*n* = 42). Twenty-one tendons from each group were incubated at 37 °C for 3.5 h, while the remaining 21 from each group were incubated for 7 h. Each group was then divided into three groups of seven, and tendon incisions were made at 25%, 50%, and 75% of the tendon thickness. Tendons were mechanically stretched axially during simultaneous collection of SWE at the injection site.

**Results:**

There were significant differences in shear wave speed (SWS) (saline > collagenase) at 3.5-h incubation (*p* < 0.001) and 7-h incubation (*p* < 0.001). Additionally, there was a significant difference in SWS between tendons cut at 25% and tendons cut at 50% and 75% (*p* = 0.040 and *p* = 0.001, respectively). Collagenase-treated tendons ruptured at a lower force than saline-treated tendons at both incubation times (both *p* < 0.001) when controlling for cut depth. Tendons treated with collagenase ruptured at a lower force than the saline control group at each cut thickness (all *p* < 0.001) controlling for incubation time.

**Conclusion:**

In a controlled *ex vivo* porcine model, SWE can be used to detect structural damage associated with tendinopathy.

**Relevance statement:**

Shear wave elastography can be used to show differences in abnormal tendons that may be translatable to clinical use as an adjunctive measure of tendon elasticity and injury.

**Key Points:**

- Tendon abnormality was quantitatively characterized using shear wave elastography in an *ex vivo* porcine experimental model.
- Shear wave speed was an accurate imaging biomarker for tendon health.
- Shear wave elastography was effective at detecting the extent of tendon damage.
- Tendons with decreased shear wave speed measurements rupture at smaller applied mechanical force.

**Graphical Abstract:**

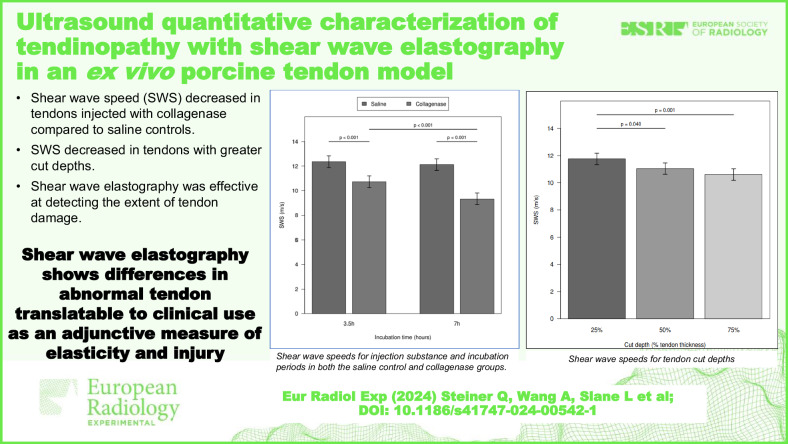

## Background

Conventional ultrasound imaging is limited to qualitative morphologic changes of tendons to diagnose tendinopathy such as tendon thickening, hypoechogenicity, and hyperemia. Although being the standard of care, conventional ultrasound suffers from significant observer variability in grading the severity of tendinopathy (*e.g*., mild, moderate, or severe) [1]. Moreover, there is a modest to high false negative and false positive rate for diagnosing tendinopathy [2].

Quantitative imaging, however, may serve as a reliable and repeatable adjunctive measure of tendon disease. Shear wave elastography (SWE) has already been utilized clinically [3, 4] and has been shown to be an accurate imaging modality with limited interobserver variability [5, 6]. These characteristics make SWE a useful imaging tool for tendinopathy, as there is a need for further improving imaging techniques for diagnosing and monitoring this condition [7]. Currently, this is done through clinical history, physical examination, and imaging studies such as magnetic resonance imaging and conventional sonography [8], which do not provide information on tendon strength, *i.e*., on its elasticity and stiffness.

Prior studies have demonstrated the effectiveness of SWE in the detection of tendinopathies through reduced tendon stiffness [9–11]. However, it has yet to be shown that SWE is capable of predicting the degree of tendon tear or ultimate tendon strength, with high spatial resolution, in a controlled tendon experimental model. In this study, we used a controlled animal model and applied 1.5% collagenase solution with and without superimposed partial tears to an *ex vivo* porcine tendon model representing tendinopathy [12]. The aim of this study was to assess the ability of SWE in quantifying tendinopathy, both tendinosis and tendon tears, in a controlled tendon model.

## Methods

This was a prospective animal tendon *ex vivo* study and was exempt from institutional review board approval. Eighty-four porcine digital flexor tendons were dissected from porcine lower limbs acquired from a local abattoir. The tendons were cleaned of muscle and connective tissue and removed from the distal bone insertion site. Tendons were divided into a test group and a control group, and then further subdivided based on incubation time and cut depth (Table 1). The *ex vivo* tendons were kept hydrated in a physiological saline solution (40 parts NaCL, 5.75 parts Na_2_HPO_4_, one part KCL, and one part KH_2_PO_4_) throughout dissection, storage (in a -30 °C freezer), and testing. For storage, the tendons were wrapped in saline-soaked gauze, then covered with aluminum foil and sealed in plastic bags before placing them in a freezer. Specimens were defrosted to room temperature before testing, one freeze-thaw cycle [13].

**Table 1:** Number of specimens in each experimental group

		Collagenase injection			Saline injection		
	Cut depth (% of total tendon width)	25	50	75	25	50	75
Incubation time	3.5 h	7	7	7	7	7	7
	7 h	7	7	7	7	7	7

Forty-two tendons were then injected with a 0.05-mL bolus of 1.5% collagenase solution to induce structural tenocyte damage and start to simulate tendinosis. The remaining 42 control tendons were injected with a 0.05-mL bolus of saline to control for the local tendon response to needle insertion and volume expansion, allowing to better elucidate what changes in SWE were strictly due to the enzymatic effect of collagenase and not the injection needle or volume. To prevent the longitudinal spread of collagenase along tendon fibers and measure focal tendinosis within a defined region of interest, two clamps were placed 5 mm apart across the central portion of the tendon. Both the collagenase-injected and control tendon groups were then divided in half, with one-half of the tendons (*i.e*., 21 per group) incubated at 37 °C for 3.5 h, the other half incubated at 37 °C for 7 h. Different incubation periods were used to represent mild and severe tendinosis, as longer incubation periods allow for greater structural tenocyte damage by the collagenase solution. In addition to tendinosis, we wanted to determine if SWE could quantify the degree of tendon damage associated with tendon tears and characterize different strain patterns based on the degree of tendon tears. Each group was then further subdivided into three groups with differing cut depths of 25%, 50%, or 75% of tendon thickness introduced to each tendon (Table 1).

### Biomechanical tests

**Fig. 1:**
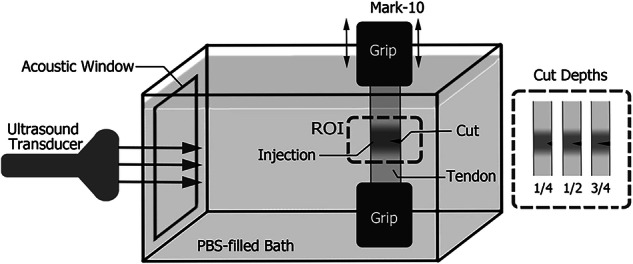
Scheme of the complete experimental setup

Prior to testing, the dimensions (*i.e*., length, width, and thickness) of the unloaded tendon were measured with a digital caliper. To measure the shear wave speed (SWS), we used a Mark-10 tensile mechanical testing system (ESM301L; Mark-10; Copiague, NY, USA) along with a custom-built water bath (Fig. 1). Consistent with previously published techniques [14], the bath was filled with a phosphate-buffered saline solution to simulate *in vivo* wave propagation between the ultrasound probe and the testing tendon. The tendon was stretched by the Mark-10 test stand, and the loading on the tendon was measured with a 50-lb load cell (M5-50; Mark-10; Copiague, NY). The preconditioning of the tensile ultimate stress strength testing was carried out by applying 0% (1.25 N preload) to 2% strain at 20 mm/min for 10 cycles. This was followed by a 1,000 s resting time before further testing.

**Fig. 2:**
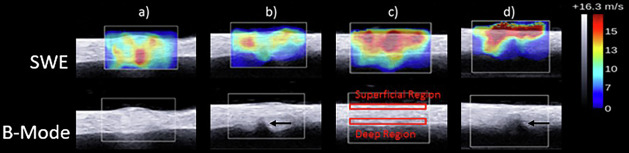
Shear wave elastography and B-mode images of porcine tendon: **a** collagenase-injected tendon; **b** collagenase-injected tendon with a partial tear at the deep region; **c** saline-injected tendon; **d** saline-injected tendon with a partial tear. Black arrows indicate the location of partial tear

In this study, the SWS maps were created to better understand the behavior of pathologic tendons and predict when tendon rupture will occur. The maps were obtained by using the Supersonic Shear Imaging on an Aixplorer® ultrasound scanner (Fig. 2) with an SL 15-4 linear array transducer (Supersonic Imagine, Aix-en-Provence, France).

The data acquisition was done in real time, as the Supersonic measures at 30,000 frames per second, thus we did not have to wait for image stabilization. The settings used in this study were: shear wave pulse (penetration, spatial smoothing) and temporal averaging (persistence) low. The SWS data were captured at the injection site with a marked region of interest of 10 × 10 mm, and acquired at 0%, 1%, and 2% strain with a 1-min resting period between each strain measurement to allow tendon fibers to recoil to a normal tension state. To introduce tear damage, we used a Thomas blade to cut the tendons to specific depths (25%, 50%, or 75% of their thickness) transversely in the deep surface away from the transducer, standardizing each cut depth by using a digital caliper and marker.

The SWS data were calculated by using a custom program developed in MATLAB (The MathWorks, Inc.; Natick, MA, USA). To determine any regional differences in SWS within the tendon, the mean values of the SWS were measured in the superficial quarter as well as in the deep quarter of the tendon (Fig. 2). Finally, the tendons were transferred to an MTS machine (MTS 858 Bionix test system with 458.20 micro-console, (MTS Systems, Eden Prairie, MN, USA)) for pull-to-failure testing to obtain the ultimate tensile stress. For each tendon tested, the mean was calculated from three repeated measurements. All measurements were performed by a researcher (A.W.) with four years of experience with these techniques.

### Data and statistical analysis

For each sample, the mean SWS was computed by averaging within the superficial and deep regions of the tendon. Force to rupture the tendon, a surrogate for tendon strength, was determined to be non-normally distributed but was sufficiently normal after logarithmic transformation. Estimates of tendon strength are reported as geometric means from exponentiation of log-based regression coefficients. Because of multiple design factors (tendon cut depth, injection media, and incubation time), the mean SWS of the region of interest at 1% strain and the ultimate tensile stress of the tendon were first analyzed with an ANOVA model with all interaction terms. The ANOVA models were then reduced to only those factors and interaction terms that significantly improved the model (*p* < 0.05). All *p*-values from statistical tests after ANOVA model reduction are appropriately adjusted for multiple testing by Holm adjustment. All adjusted *p*-values are considered significant at a 0.05 significance level. Analysis was conducted using R for statistical computing version 4.1.

## Results

The three-way ANOVA model with average SWS as the outcome was reduced to all three individual factors (injection, incubation time, and cut depth) plus the interaction between injection and incubation time (*p* = 0.017). The tendon strength ANOVA model was reduced to the three factors plus interaction terms for injection and incubation time (*p* < 0.001) and injection and cut depth (*p* < 0.001). The results of the ANOVA models are presented in Supplementary Table 1.

### Average SWS and cut depth

There was no significant injection:cut depth interaction (*p* = 0.065). However, when controlling for injection type and time, a larger cut depth was associated with a decrease in SWS. There was a significant difference in SWS between tendons cut at 25% and tendons cut at 50% and 75% (*p* = 0.040 and *p* = 0.001, respectively). No difference was noted in SWS between tendons cut at 50% and 75% (*p* = 0.149). Measurement values are shown in Table 2.

**Table 2:** Average SWS measurements for each cut depth with statistical comparisons

Cut depth	Mean (m/s)	95% confidence interval
25%	11.8	(11.3–12.2)
50%	11.0	(10.6–11.5)
75%	10.6	(10.2–11.0)

Cut depth comparison	*p*-value	
25% *versus* 50%	**0.040**	
25% *versus* 75%	**0.001**	
50% *versus* 75%	0.149	

Statistically significant *p*-values (< 0.05) are bolded

### Average SWS and incubation time

**Fig. 3:**
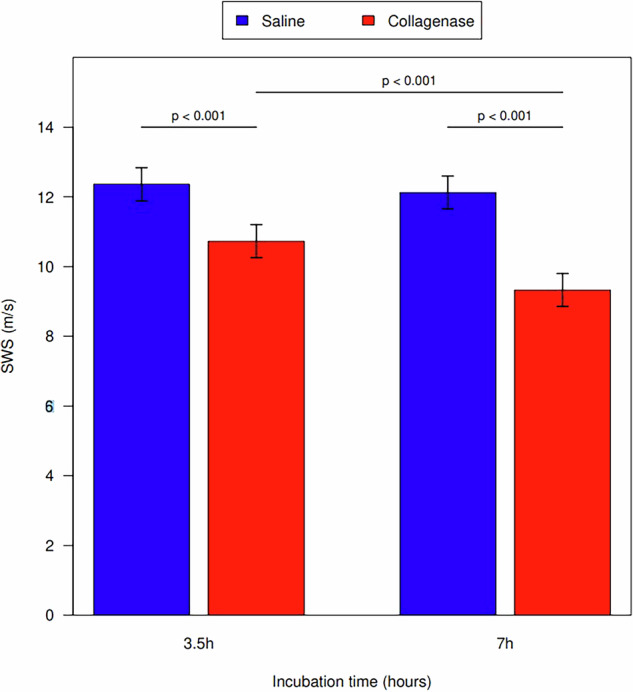
Shear wave speeds for injection substance and incubation periods in both the saline control and collagenase groups

Specimens incubated in collagenase had reduced average SWS compared with the saline control group when controlling for cut depth (*p* < 0.001 for 3.5-h incubation; *p* < 0.001 for 7-h incubation). A longer incubation time was associated with a reduction in SWS between the collagenase groups (*p* < 0.001), but not in the saline control group. (*p* = 0.481) (Fig. 3). Measurement values are shown in Table 3.

**Table 3:** Mean tendon SWS for each incubation time and injection type

Time (h)	Injection type	SWS (m/s)		Saline *minus* Collagenase	3.5 *minus* 7 h
		Mean	SD	Diff. (95% CI)	Diff. (95% CI)
3.5	Collagenase	10.73	1.05		1.40 (0.73–2.07)
7	Collagenase	9.33	1.04		
3.5	Saline	12.36	1.17	1.63 (0.96–2.30)	0.24 (-0.43 to 0.91)
7	Saline	12.12	1.43	2.80 (2.13–3.47)	

*CI* Confidence interval, *SD* Standard deviation, *SWS* Shear wave speed

### Tendon strength and incubation time

**Fig. 4:**
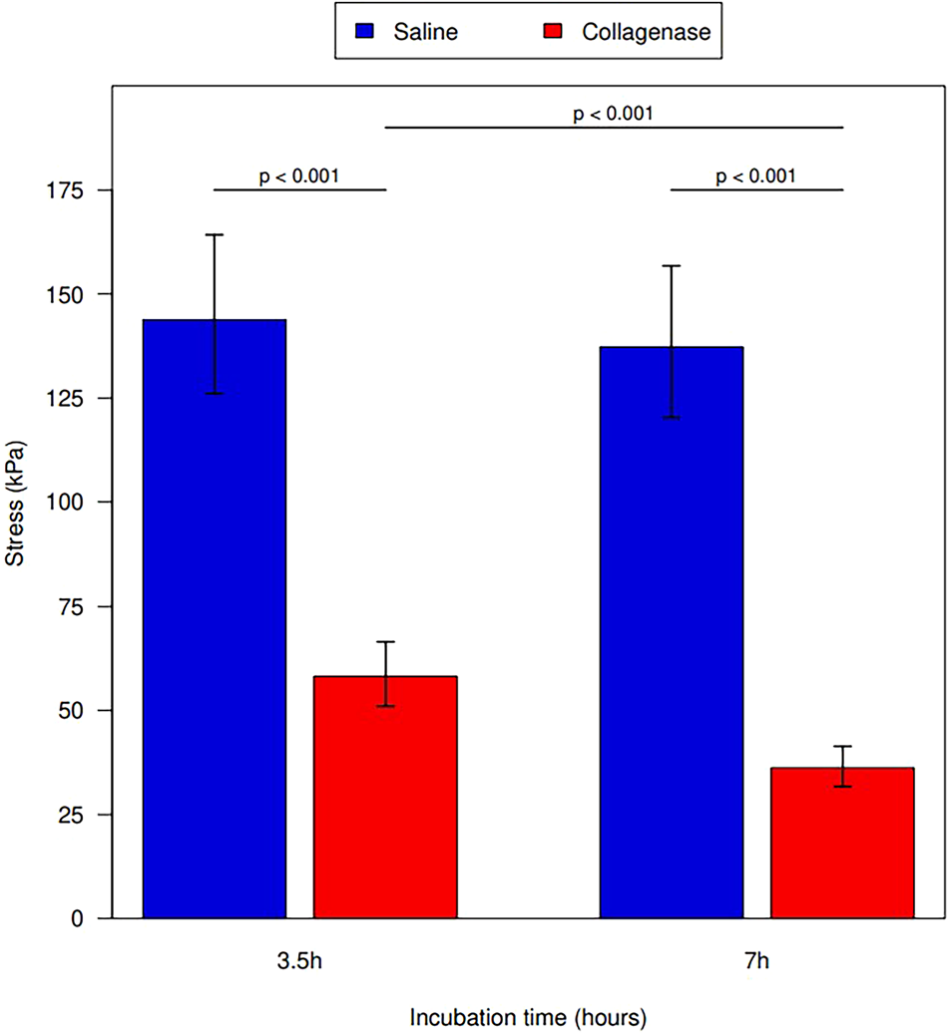
Ultimate tendon stress for incubation time in both the saline control and collagenase groups

While controlling for cut depth, saline-treated tendons required a significantly higher amount of force to rupture when compared to collagenase-treated tendons at both 3.5 h of incubation (*p* < 0.001) and 7 h of incubation (*p* < 0.001) (Fig. 4). Tendons incubated in collagenase for 7 h required a significantly less amount of force to rupture compared to collagenase tendons incubated for 3.5 h (*p* < 0.001). Saline-treated specimens did not have a significant change in tendon strength based on incubation time (*p* = 0.622). Measurement values are shown in Table 4.

**Table 4:** Geometric mean tensile strength for each incubation time and injection type

Time (h)	Injection type	Tensile strength (N/s^2^)		Saline to collagenase	3.5 to 7 h
		Geometric mean	SD	Log ratio (95% CI)	Log ratio (95% CI)
3.5	Collagenase	58.2	24.6		1.61 (1.33–1.94)
7	Collagenase	36.2	9.5		
3.5	Saline	143.8	84.9	2.47 (2.05–2.98)	1.05 (0.87–1.26)
7	Saline	137.3	73.2	3.79 (3.14–4.57)	

*CI* Confidence interval, *SD* Standard deviation

### Tendon strength and cut depth

**Fig. 5:**
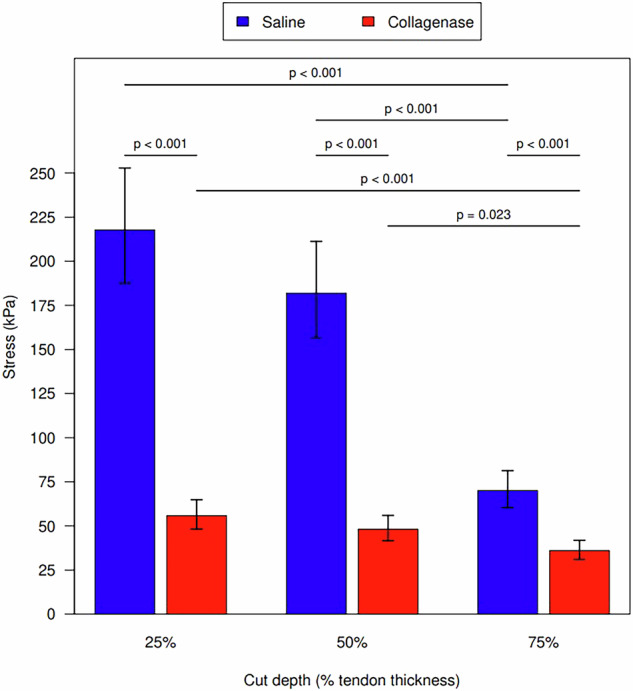
Ultimate tendon stress for cut depths in both the saline control and collagenase groups

Controlling for incubation time, tendons treated with collagenase had less tendon strength than the saline control group at 25%, 50%, and 75% cut depth (all *p* < 0.001). In both saline and collagenase groups, progressive decreases in tendon strength occurred at each increase in cut depth. The decrease in tendon strength was not statistically significant from 25% to 50% cut depth (saline *p* = 0.188; collagenase *p* = 0.188). The decrease was statistically significant from 25% to 75% (both *p* < 0.001) and from 50% to 75% cut depth (saline *p* < 0.001; collagenase *p* = 0.023) (Fig. 5). Measurement values are shown in Table 5.

**Table 5:** Geometric mean tendon tensile strength for each cut depth and injection type

Cut percentage	Injection type	Tensile strength (N/m^2^)		Saline to collagenase	Cut percentage comparison within Injection type	
		Geometric mean	SD	Log ratio (95% CI)	Comparison	Log ratio (95% CI)
25	Collagenase	55.8	17.94		25 to 50	1.16 (0.90–1.49)
50	Collagenase	48.2	29.60		25 to 75	1.55 (1.20–2.00)
75	Collagenase	36.0	10.02		50 to 75	1.34 (1.04–1.72)
25	Saline	217.8	60.35	3.90 (3.16–4.82)	25 to 50	1.20 (0.93–1.54)
50	Saline	182.0	43.59	3.78 (3.06–4.66)	25 to 75	3.11 (2.41–4.01)
75	Saline	70.0	15.91	1.94 (1.57–2.40)	50 to 75	2.60 (2.02–3.35)

*CI* Confidence interval, *SD* Standard deviation

## Discussion

In our study, we found that collagenase-injected tendons had decreased SWS when compared to the control tendons. This result is in line with our expectations based on the pathophysiology of tendinosis and previous work done by Quilling et al [11]. Additionally, this reduction in SWS was time-dependent as there was a further reduction in SWS after 7 h of incubation in comparison to 3.5 h. These results suggest SWE can identify damage within a tendon and quantify the level of damage as the SWS was significantly lower in tendons that were allowed to incubate for a longer period, simulating moderate to severe tendinosis *versus* mild tendinosis at 3.5 h incubation.

Our results associated with SWS and cut depth were in line with recent work published by DeWall et al examining SWS in association with tendon cut depth [15]. We found that a greater cut depth was associated with decreased SWS measurements, again suggesting that SWE can help quantify the level of tendon damage. Additionally, although larger cut depths were associated with decreased SWS, we did not see a stepwise decrease with increasing cut depth. This could suggest that SWE is useful in differentiating small tendon tears and deep partial thickness tears but less able to differentiate between varying degrees of deep partial tendon tears. This supports the clinical utility of SWE for identifying minor tears from clinically relevant deep partial thickness tears of > 50% of the tendon width. Interestingly, we did not find any interaction between injection type and cut depth. Examining cut depth and collagenase injections concomitantly, should simulate the clinical presentation of a tendon tear superimposed on tendinosis [15]. These findings may suggest that SWS may not be as clinically useful in diagnosing tendinosis in patients with concomitant tendon tears.

For our ultimate stress testing, we found collagenase-injected tendons showed significantly reduced ultimate stress in comparison to the controls regardless of cut depth. The saline control group showed a stepwise decrease in ultimate stress at each cut depth. This may suggest that collagenase degrades the tendon enough that changes in cut depth are no longer detectable. However, these findings support our hypothesis and previous studies that tendons with decreased SWS, and therefore decreased elasticity, would be expected to rupture at lower pull forces [11].

Given the results of our study and previous literature [16–18], SWE may be an effective imaging tool to quantitatively measure the extent of tendon disease and improve the current diagnostic approach. However, there are still additional obstacles to its clinical translation as current published studies are pre-clinical, and there are still insufficient clinical feasibility studies [19]. Additionally, although SWE has been shown to be a more reliable quantitative imaging technique in other organs, such as the breast and liver, there are still limitations on the reproducibility of SWE values in tendons [20]. However, if future *in vivo* clinical studies demonstrate that SWE provides a quantitative measure of tendon disease, then we may be able to prevent tendon rupture by routine screening and implementation of activity modifications in at-risk patients. This animal model validation study is the first step toward this goal.

This study has limitations. There were seven porcine flexor tendons in each treatment group. Because of the large number of groups, we were still able to make meaningful conclusions, but a larger number of tendons in each group, or a smaller number of groups would have improved the study validity. Although we used saline-injected tendons to control for needle damage we did not test any tendons without any injections—the addition of volume may have an effect on tendon stiffness that we did not measure. Furthermore, we did not analyze differences based on the interval between the pig’s death and tendon processing. We did not perform repeatability analysis on this experiment, given that SWE has already been established as repeatable in previous studies [21]. Furthermore, an *ex vivo* model may not correlate perfectly with *in vivo* results. However, our results will help inform future *in vivo* study designs.

In conclusion, in a controlled *ex vivo* porcine model, SWE was able to detect structural damage associated with experimental abnormalities, showing a potential for clinical quantitative measure of tendon injury and elasticity. Future studies with large sample sizes are needed to allow for more robust comparisons. Additionally, studies using SWE to measure tendons in humans are needed to account for factors in *in vivo* tendons that our study did not.

## Supplementary information

**Additional file 1:** **Supplemental** Table 1. Results of ANOVA models for average SWS and tendon force to rupture.

## Data Availability

The datasets used and/or analyzed during the current study are available from the corresponding author upon reasonable request.

## Abbreviations

SWE: Shear wave elastography
SWS: Shear wave speed
