# Supplementary material for: Ultrasound quantitative characterization of tendinopathy with shear wave elastography in an ex vivo porcine tendon model
Source: Eur Radiol Exp. 2025 Mar 20;9:33. doi: 10.1186/s41747-024-00542-1 (PMC11926283; doi:10.1186/s41747-024-00542-1)
Supplement: Supplementary file 1 — Additional file 1: Supplemental Table 1. Results of ANOVA models for average SWS and tendon force to rupture. [file 41747_2024_542_MOESM1_ESM.pdf]

Ultrasound quantitative characterization of tendinopathy with shear wave elastography in an ex vivo porcine tendon model
ELECTRONIC SUPPLEMENTARY MATERIAL

Supplemental Table 1. Results of ANOVA models for average SWS and tendon force to rupture.

Average Shear wave speed	
	p-value
Injection Type	**< 0.001**
Incubation Time	**0.001**
Cut Depth	**0.001**
Injection:Incubation Time	**0.017**
Force to rupture	
	p-value
Injection Type	**< 0.001**
Incubation Time	**< 0.001**
Cut Depth	**< 0.001**
Injection:Incubation Time	**< 0.001**
Injection:Cut Depth	**< 0.001**
